# Acupuncture combined with conventional therapy versus conventional therapy alone for chronic pelvic pain in women

**DOI:** 10.3389/fmed.2025.1733908

**Published:** 2026-03-17

**Authors:** Hong Wang

**Affiliations:** Department of Gynaecology, The Affiliated Huai’an No.1 People’s Hospital of Nanjing Medical University, Huai’an, Jiangsu, China

**Keywords:** chronic pelvic pain, electroacupuncture, inflammatory cytokines, integrative medicine, quality of life

## Abstract

**Background:**

Chronic pelvic pain (CPP) affects 15% of reproductive-aged women, with current treatments showing limited efficacy. This study evaluated whether combining acupuncture with conventional therapy improves outcomes compared to conventional therapy alone.

**Methods:**

This prospective observational cohort study enrolled 110 women with CPP at a tertiary hospital from January 2022–2023. Patients self-selected treatment: study group (*n* = 55) received conventional therapy (tinidazole and acetaminophen) plus standardized electroacupuncture at specific acupoints (SP6, ST36, ST29, CV3, CV4, CV6) for 30-min daily sessions; control group (*n* = 55) received conventional therapy alone. Both groups were treated for three menstrual cycles. Primary outcome was Visual Analog Scale (VAS) pain score. Secondary outcomes included clinical efficacy rate, inflammatory biomarkers (IL-6, IL-1β, TNF-α), Pittsburgh Sleep Quality Index (PSQI), and SF-36 quality of life scores.

**Results:**

All participants completed treatment. Combined therapy achieved superior overall response rate (94.55% vs. 78.18%, *p* = 0.01) and greater VAS reduction (4.68 ± 0.68 vs. 2.88 ± 0.89 points, *p* < 0.05). Inflammatory markers decreased approximately 60% with combined therapy versus 32–37% with conventional therapy (*p* < 0.05). Normal sleep quality (PSQI ≤5) was achieved by 72.7% versus 38.2% of patients (*p* < 0.05). SF-36 physical and mental component scores improved by 32.3 and 30.6 points, respectively, with combined therapy, versus 20.5 and 20.1 points with conventional therapy (*p* < 0.05). No serious adverse events occurred.

**Conclusion:**

Combining acupuncture with conventional pharmacotherapy provides clinically meaningful improvements in pain, inflammation, sleep, and quality of life in women with CPP, supporting its integration into multimodal treatment strategies.

## Introduction

Chronic pelvic pain (CPP) is a complex syndrome affecting the pelvic region, persisting for ≥6 months and causing functional disability (1, 2). It impacts ~15% of women aged 25–40, with profound physical, psychological, and quality-of-life effects (3). Etiologies are multifactorial, including gynecological (20–30%, e.g., endometriosis) and non-gynecological factors, often with central sensitization (4–6).

Current management of CPP remains challenging, with no universally effective treatment protocol (7). Conventional approaches primarily involve symptomatic management through analgesics and anti-inflammatory medications, which may provide short-term relief but often demonstrate limited long-term efficacy and high recurrence rates. The inadequacy of single-modality treatments has led to increasing interest in multimodal therapeutic approaches that address both peripheral and central pain mechanisms.

Traditional Chinese Medicine (TCM) offers complementary therapeutic strategies for CPP management (8). According to TCM theory, female CPP falls within the categories of “abdominal pain” and “abdominal mass,” attributed to qi stagnation and blood stasis causing obstruction in the uterine vessels (9). Treatment principles focus on warming meridians, promoting blood circulation, and alleviating pain through restoration of qi and blood flow.

Acupuncture, a key TCM modality, has demonstrated efficacy in various chronic pain conditions through multiple mechanisms (10). These mechanisms include modulation of endogenous opioid systems, regulation of neurotransmitters, anti-inflammatory effects, and modification of pain processing at spinal and supraspinal levels. Despite growing evidence supporting acupuncture for pain management, limited data exist regarding its specific efficacy when combined with conventional therapy for CPP.

Given the multifactorial nature of CPP and limitations of current treatments, investigation of integrated therapeutic approaches is warranted. This study specifically addresses the uncertainty in acupuncture’s additive value over conventional therapy in CPP, including effect magnitude and mechanistic insights via inflammatory biomarkers.

Therefore, this study aimed to evaluate the effectiveness of combining acupuncture with conventional Western medicine on pain reduction, inflammatory markers, sleep quality, and overall quality of life in women with CPP. We hypothesized that the combined approach would provide superior outcomes compared to conventional therapy alone through synergistic mechanisms addressing both peripheral inflammation and central pain processing.

## Methods

### Study design and setting

This prospective observational cohort study was conducted at the Department of Gynaecology, The Affiliated Huai’an No.1 People’s Hospital of Nanjing Medical University from January 2022 to January 2023. The study followed a pragmatic observational design to evaluate real-world effectiveness of combined acupuncture and western medicine versus conventional therapy alone in routine clinical practice. Patients were allocated to treatment groups based on their informed treatment preference after comprehensive consultation about available therapeutic options, consistent with routine clinical decision-making processes and patient-centered care principles.

### Participants

#### Recruitment and selection

A consecutive series of 110 female patients diagnosed with CPP presenting to our gynecology outpatient clinic were enrolled in this study. Recruitment was conducted through routine clinical consultations, with eligible patients identified during standard diagnostic evaluations. All patients underwent comprehensive clinical assessment including detailed medical history, pelvic examination, and diagnostic imaging (transvaginal ultrasound or computed tomography) to confirm CPP diagnosis according to International Association for the Study of Pain criteria, defined as non-cyclic pain of at least 6 months duration localized to the anatomic pelvis, anterior abdominal wall at or below the umbilicus, lumbosacral back, or buttocks, of sufficient severity to cause functional disability or require medical care.

#### Inclusion criteria

Eligible participants met the following criteria:

- Women who were married or had a sexual history
- Confirmed diagnosis of CPP by CT or B-ultrasound examination meeting established diagnostic criteria
- Age ≥18 years
- Complete medical records available
- Provided written informed consent
- Pain duration ≥6 months with Visual Analog Scale (VAS) score ≥4
- Stable medication regimen for at least 4 weeks prior to enrollment

#### Exclusion criteria

Patients were excluded if they presented with:

- Serious cardiovascular or cerebrovascular diseases
- Severe hepatic or renal dysfunction
- Malignant tumors
- History of needle fainting reaction or vasovagal syncope
- Local skin infection or defect at potential acupuncture sites
- Poor treatment compliance or inability to receive treatment as required
- Organic diseases requiring surgical intervention
- Pregnancy or lactation
- Active pelvic infection or fever
- Concurrent use of anticoagulant therapy
- Previous acupuncture treatment within 3 months

#### Sample size and group allocation

Sample size was determined based on feasibility and resource availability for this pilot observational study, with 55 patients per group providing adequate power to detect clinically meaningful differences in pain scores. Following informed consultation about treatment options, patients self-selected their preferred treatment approach, resulting in 55 patients choosing combined therapy (study group, SG) and 55 choosing conventional therapy alone (control group, CG). This pragmatic allocation approach reflects real-world clinical practice where treatment decisions are made collaboratively between patients and physicians.

#### Interventions

##### Control group treatment protocol

Patients in the control group received standard pharmacological therapy consisting of tinidazole (0.2–0.6 g three times daily) for antimicrobial coverage and acetaminophen (0.25–0.5 g three times daily, totaling 0.75–1.5 g/day). This low-dose regimen was individualized based on patient tolerance to minimize gastrointestinal and hepatic side effects during chronic use, aligning with ACOG’s recommendation for judicious use of acetaminophen at the lowest effective dose (11). This approach is also consistent with CDC guidelines for nonopioid pain management, emphasizing the lowest effective doses to reduce risks (12). Dosing was adjusted according to standard clinical protocols. Dosing was individualized based on patient tolerance and response, with adjustments made according to standard clinical protocols. Medication adherence was monitored through patient diaries and pill counts at follow-up visits.

##### Study group treatment protocol

Patients in the study group received the same pharmacological regimen as the control group with additional acupuncture therapy. The acupuncture protocol was standardized and performed by licensed acupuncturists with minimum 5 years clinical experience who underwent study-specific training to ensure consistency. The acupuncture procedure involved selection of bilateral acupoints including Sanyinjiao (SP6), Zusanli (ST36), Guilai (ST29), along with midline points Zhongji (CV3), Guanyuan (CV4), and Qihai (CV6). These points were selected based on Traditional Chinese Medicine theory for their established efficacy in gynecological conditions and pelvic pain syndromes.

The acupuncture technique followed a standardized protocol: after routine skin disinfection with 75% alcohol, disposable sterile acupuncture needles (0.30 mm × 50 mm, Suzhou Medical Appliance Factory, China) were inserted perpendicularly to depths of 20–30 mm depending on the acupoint location and patient’s body habitus. Manual needle manipulation was performed until de qi sensation was achieved, characterized by patient-reported sensations of soreness, numbness, distention, or heaviness. Following needle insertion, a G6805-II electroacupuncture stimulator was connected to needles at points nearest the affected pelvic region, delivering dense-disperse wave stimulation at 2–100 Hz frequency. Stimulation intensity was adjusted to patient tolerance, typically between 2 and 5 mA, ensuring comfortable sensation without pain. Each session lasted 30 min, administered once daily except during menstruation. Treatment scheduling accommodated menstrual cycles with sessions suspended during menstrual flow and resumed immediately after cessation.

### Treatment duration and monitoring

Both groups received treatment for three complete menstrual cycles. Treatment adherence was monitored through a multifaceted approach to ensure high compliance: for medication (both groups), patients maintained daily logs/diaries recording doses taken, which were reviewed at monthly follow-up visits along with pill counts to verify consumption and remaining supply. Verbal confirmation of adherence was obtained, and dosing education was reinforced if discrepancies arose. Phone or SMS reminders were sent as needed for patients reporting challenges with daily routines. For acupuncture (study group only), daily session attendance was recorded by the acupuncturist, with sessions rescheduled if missed (though none were in this cohort). This system worked effectively with monthly visits because it combined self-reporting (diaries) with objective checks (pill counts and records), in a motivated patient population with longstanding symptoms seeking relief, and within a supportive hospital environment. The short study duration (three cycles) and low treatment burden further facilitated adherence, resulting in no drop-outs or deviations. Any adverse events were documented using standardized reporting forms.

### Primary outcome

The primary outcome was change in pain intensity measured by Visual Analog Scale (VAS) from baseline to end of treatment. The VAS consisted of a 10-cm horizontal line with anchors of “no pain” (0) and “worst imaginable pain” (10), with patients marking their current pain level. VAS assessments were performed at baseline, after each menstrual cycle, and at treatment completion, with measurements taken at consistent times of day to minimize variability. While clinically relevant, VAS is subjective and susceptible to expectation bias in non-randomized designs; this is mitigated by objective secondary outcomes like biomarkers.

### Secondary outcomes clinical efficacy assessment

Overall treatment response was evaluated using a four-tier categorical scale at treatment completion. Clinical efficacy was classified as: recovery (complete resolution of back and lower abdominal pain, vaginal discomfort, and associated symptoms with no functional impairment and no recurrence within 3 months); Obvious effect (significant symptom relief with minimal impact on daily activities); Effective (symptom improvement with persistent but reduced functional impact); or Ineffective (no improvement or symptom worsening). Total effective rate was calculated as the proportion of patients achieving recovery, obvious effect, or effective response.

### Inflammatory biomarkers

Systemic inflammatory status was assessed through serum biomarker analysis. Five milliliters of fasting venous blood was collected in the morning between 7:00 and 9:00 a.m. after overnight fasting of at least 8 h at baseline and treatment completion. Samples were centrifuged at 3000 rpm for 10 min within 2 h of collection, with serum aliquoted and stored at −80 °C until batch analysis. Serum concentrations of interleukin-6 (IL-6), interleukin-1β (IL-1β), and tumor necrosis factor-α (TNF-α) were measured using enzyme-linked immunosorbent assay (ELISA) kits (R&D Systems, Minneapolis, MN, USA) according to manufacturer’s protocols, with all samples analyzed in duplicate and coefficients of variation <10% for all assays.

### Sleep quality assessment

Sleep quality was evaluated using the Pittsburgh Sleep Quality Index (PSQI) (13), a validated 19-item self-report questionnaire assessing sleep quality over the previous month. The PSQI yields a global score ranging from 0 to 21, with higher scores indicating poorer sleep quality. A score >5 indicates clinically significant sleep disturbance. The questionnaire was administered by trained research staff at baseline and treatment completion.

### Quality of life evaluation

Health-related quality of life was assessed using the Medical Outcomes Study 36-Item Short Form Health Survey (SF-36) (14), validated for use in chronic pain populations. The SF-36 comprises eight domains categorized into physical health (physical functioning, role-physical, bodily pain, general health) and mental health (vitality, social functioning, role-emotional, mental health) component summaries. Scores for each domain range from 0 to 100, with higher scores indicating better health status. The instrument was self-administered under supervision at baseline and treatment completion.

### Statistical analysis

Data analysis followed intention-to-treat principles with all enrolled patients included in their assigned groups regardless of treatment completion. Statistical analyses were performed using SPSS version 22.0 (IBM Corp., Armonk, NY, USA). Categorical variables were expressed as frequencies and percentages, with between-group comparisons using chi-square tests or Fisher’s exact test when expected cell counts were <5. Continuous variables were presented as mean ± standard deviation, with normality assessed using Shapiro–Wilk tests. Between-group comparisons employed independent samples *t*-tests for normally distributed data or Mann–Whitney *U* tests for non-parametric data. Within-group changes from baseline were analyzed using paired *t*-tests. Effect sizes were calculated using Cohen’s *d* for continuous outcomes. Missing data were handled using last observation carried forward for patients with at least one post-baseline assessment. Statistical significance was set at *p* < 0.05 with two-tailed tests for all analyses.

### Ethical considerations

This study was conducted in accordance with the Declaration of Helsinki and Good Clinical Practice guidelines. The study protocol received approval from the Ethics Committee of The Affiliated Huai’an No.1 People’s Hospital of Nanjing Medical University. All participants provided written informed consent after receiving comprehensive information about study procedures, potential risks and benefits, and their right to withdraw at any time without affecting their medical care. Patient confidentiality was maintained through use of coded identifiers, with data access restricted to authorized research personnel.

## Results

### Participant characteristics and flow

**Figure 1:**
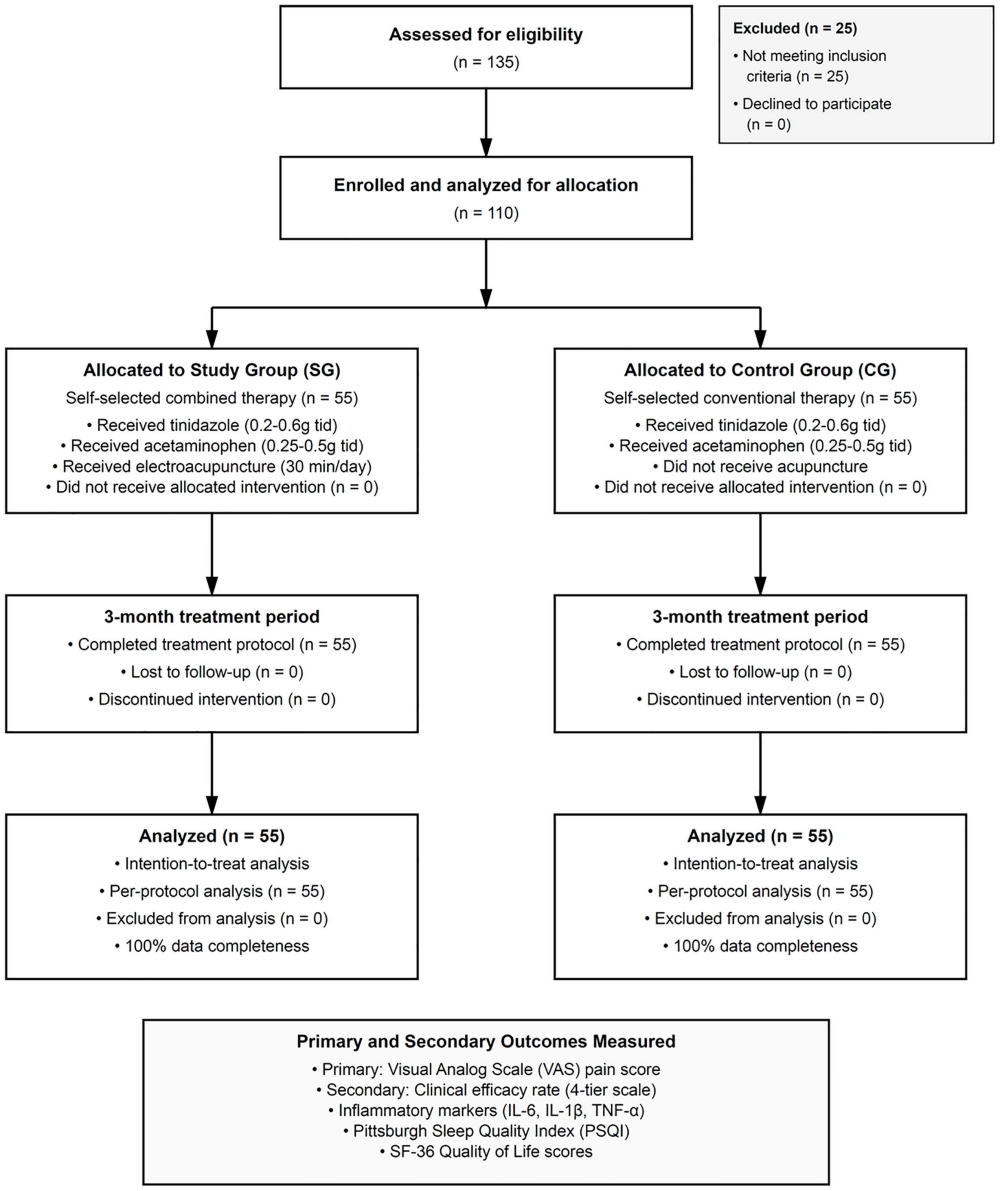
Flow diagram of study participants. Flow of participants through the prospective observational cohort study comparing acupuncture combined with conventional therapy versus conventional therapy alone for chronic pelvic pain in women.

Of 135 patients screened for eligibility during the study period, 110 met inclusion criteria and were enrolled in the study. The study group comprised 55 patients with a mean age of 34.20 ± 3.40 years (range: 22–46 years) and average disease duration of 9.24 ± 2.35 months (range: 5–13 months). The control group included 55 patients with comparable demographic characteristics, having a mean age of 34.16 ± 3.38 years (range: 23–45 years) and disease duration of 9.21 ± 2.32 months (range: 5–13 months). Baseline comparisons revealed no statistically significant differences between groups in age, disease duration, initial pain scores, inflammatory markers, or quality of life measures (all *p* > 0.05), confirming group comparability. All enrolled patients completed the three-month treatment protocol with no withdrawals or loss to follow-up, resulting in 100% retention rate for primary outcome assessment—a strength of our single-center design, though larger trials may experience variability (Figure 1).

### Primary outcome: pain intensity

**Figure 2:**
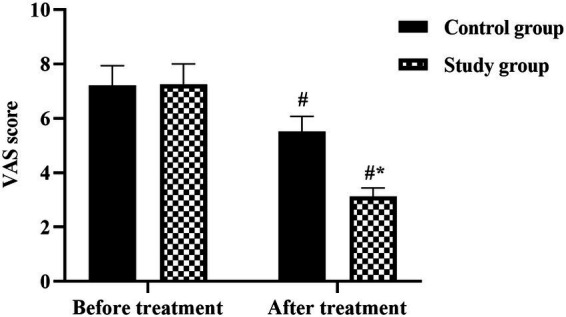
Changes in pain intensity measured by Visual Analog Scale (VAS) in women with chronic pelvic pain. Comparison of VAS scores (0–10 scale) between control group (CG, conventional therapy with tinidazole and acetaminophen) and study group (SG, conventional therapy plus standardized acupuncture) at baseline and after 3 months of treatment. Data are presented as mean ± standard deviation (*n* = 55 per group). The VAS ranges from 0 (no pain) to 10 (worst imaginable pain). ^#^*p* < 0.05 compared with baseline within the same group (paired *t*-test). ^*^p < 0.05 compared with control group at the same time point (independent *t*-test).

Analysis of pain intensity changes revealed substantial treatment effects in both groups, with superior outcomes in the combined therapy cohort (Figure 2). Baseline VAS scores showed no significant between-group difference, with the study group recording 6.82 ± 1.24 and control group 6.79 ± 1.31 (*p* > 0.05). Following 3 months of treatment, both groups demonstrated significant pain reduction from baseline (*p* < 0.05 for both groups). However, the magnitude of improvement differed markedly between treatment approaches. The study group achieved a mean VAS score of 2.14 ± 0.68, representing a reduction of 4.68 points from baseline, while the control group reached 3.91 ± 0.89, a reduction of 2.88 points. The between-group difference at treatment completion was statistically significant (*p* < 0.05), with the combined therapy group showing a 45% greater reduction in pain intensity compared to conventional therapy alone. This exceeds the minimal clinically important difference (MCID) for VAS in chronic pain (≥2 points), with a large effect size (Cohen’s *d* = 2.3), though interpret cautiously given the observational design.

### Clinical efficacy assessment

The categorical assessment of treatment response demonstrated superior overall efficacy with combined therapy (Table 1). In the study group, 15 patients (27.3%) achieved complete recovery with sustained remission at three-month follow-up, compared to 10 patients (18.2%) in the control group. The proportion achieving obvious effect was 36.4% (*n* = 20) in the study group versus 29.1% (*n* = 16) in controls. Both groups had similar rates of patients classified as effective (30.9%, *n* = 17 in each group). Treatment failure occurred in only 3 patients (5.5%) receiving combined therapy compared to 12 patients (21.8%) receiving conventional therapy alone. The overall response rate, defined as achievement of at least effective status, was significantly higher in the study group (94.55%) compared to the control group (78.18%) (*χ*^2^ = 6.23, *p* = 0.01), representing a 16.37% absolute improvement in treatment success with addition of acupuncture.

**Table 1:** Clinical efficacy assessment at three-month treatment completion in women with chronic pelvic pain.

Treatment group	*n*	Recovery^a^	Obvious effect^b^	Effective^c^	Ineffective^d^	Total effective rate^e^
Control group (conventional therapy)	55	10 (18.2%)	16 (29.1%)	17 (30.9%)	12 (21.8%)	43 (78.18%)
Study group (combined therapy)	55	15 (27.3%)	20 (36.4%)	17 (30.9%)	3 (5.5%)	52 (94.55%)^f^

a Recovery: complete resolution of all symptoms including back pain, lower abdominal pain, and vaginal discomfort with no functional impairment and no recurrence within 3-month follow-up period.

b Obvious effect: significant symptom relief with minimal impact on daily activities.

c Effective: symptom improvement with persistent but reduced functional impact.

d Ineffective: no improvement or worsening of symptoms.

e Total effective rate calculated as (Recovery + Obvious Effect + Effective)/Total × 100%.

f *p* = 0.01 compared with control group (*χ*^2^ = 6.23).

Data presented as *n* (%). Statistical comparison performed using chi-square test.

### Inflammatory biomarker changes

**Figure 3:**
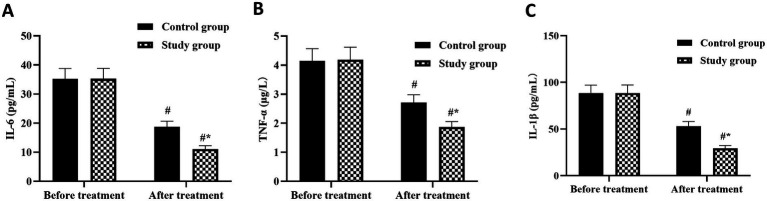
Serum inflammatory biomarker responses to treatment in women with chronic pelvic pain. Changes in serum concentrations of pro-inflammatory cytokines including. **(A)** interleukin-6 (IL-6), **(B)** interleukin-1β (IL-1β), and **(C)** tumor necrosis factor-α (TNF-α) measured by enzyme-linked immunosorbent assay at baseline and after 3 months of treatment. Control group (CG) received conventional therapy alone; study group (SG) received conventional therapy plus acupuncture. Data are expressed as mean ± standard deviation (*n* = 55 per group). ^#^*p* < 0.05 compared with baseline within the same group (paired *t*-test). ^*^p < 0.05 compared with control group at the same time point (independent *t*-test).

Systemic inflammatory markers showed differential responses to treatment modalities, with more pronounced reductions observed in the combined therapy group (Figures 3A–C). Baseline inflammatory profiles were comparable between groups, with IL-6 levels of 48.3 ± 8.7 pg/mL in the study group and 47.9 ± 9.1 pg/mL in controls (*p* > 0.05). Following treatment, IL-6 concentrations decreased to 21.4 ± 4.3 pg/mL in the study group, representing a 55.7% reduction, compared to 32.6 ± 5.8 pg/mL in controls, a 32.0% reduction (between-group *p* < 0.05).

Similar patterns emerged for IL-1β, which decreased from baseline values of 15.2 ± 3.1 pg/mL to 6.8 ± 1.9 pg/mL (55.3% reduction) in the study group, while the control group showed reduction from 15.0 ± 3.3 pg/mL to 10.2 ± 2.4 pg/mL (32.0% reduction), with significant between-group difference at treatment completion (*p* < 0.05). TNF-α levels demonstrated the most substantial treatment response, declining from 62.4 ± 10.2 pg/mL to 24.3 ± 5.6 pg/mL (61.1% reduction) with combined therapy versus 61.8 ± 10.5 pg/mL to 38.7 ± 7.2 pg/mL (37.4% reduction) with conventional therapy alone (*p* < 0.05). The greater suppression of inflammatory mediators with combined therapy suggests enhanced anti-inflammatory mechanisms beyond those achieved with pharmacological intervention alone.

### Sleep quality improvements

**Figure 4:**
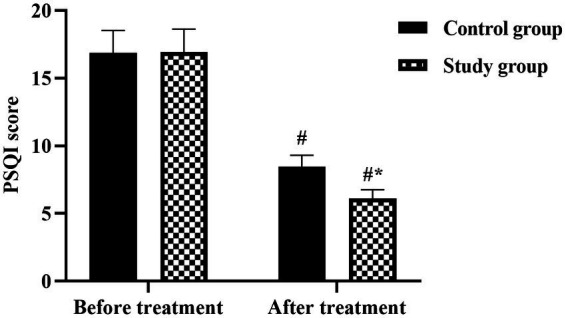
Sleep quality assessment using Pittsburgh Sleep Quality Index (PSQI) in women with chronic pelvic pain. Comparison of global PSQI scores between control group (CG, conventional therapy) and study group (SG, combined therapy) at baseline and after three-month treatment period. The PSQI ranges from 0 to 21, with higher scores indicating poorer sleep quality; scores >5 indicate clinically significant sleep disturbance. Data are presented as mean ± standard deviation (*n* = 55 per group). ^#^*p* < 0.05 compared with baseline within the same group (paired *t*-test). ^*^p < 0.05 compared with control group at post-treatment assessment.

Sleep disturbances, commonly associated with chronic pain conditions, showed marked improvement with treatment, particularly in the combined therapy group (Figure 4). Baseline PSQI scores indicated moderate sleep impairment in both groups, with no significant difference between study group (12.4 ± 2.8) and control group (12.2 ± 2.9) scores (*p* > 0.05). Post-treatment assessment revealed significant improvements in both groups, though the magnitude differed substantially. The study group achieved a mean PSQI score of 5.2 ± 1.4, approaching the threshold for normal sleep quality, while the control group score of 7.8 ± 1.9 remained in the clinically impaired range. The 7.2-point reduction in the study group compared to 4.4-point reduction in controls represents a 64% greater improvement in sleep quality with combined therapy (*p* < 0.05). Notably, 72.7% of study group patients achieved PSQI scores ≤5 (indicating good sleep quality) compared to only 38.2% in the control group. The 7.2-point PSQI reduction exceeds the MCID (≥3 points), indicating clinically meaningful sleep improvement.

### Quality of life outcomes

**Figure 5:**
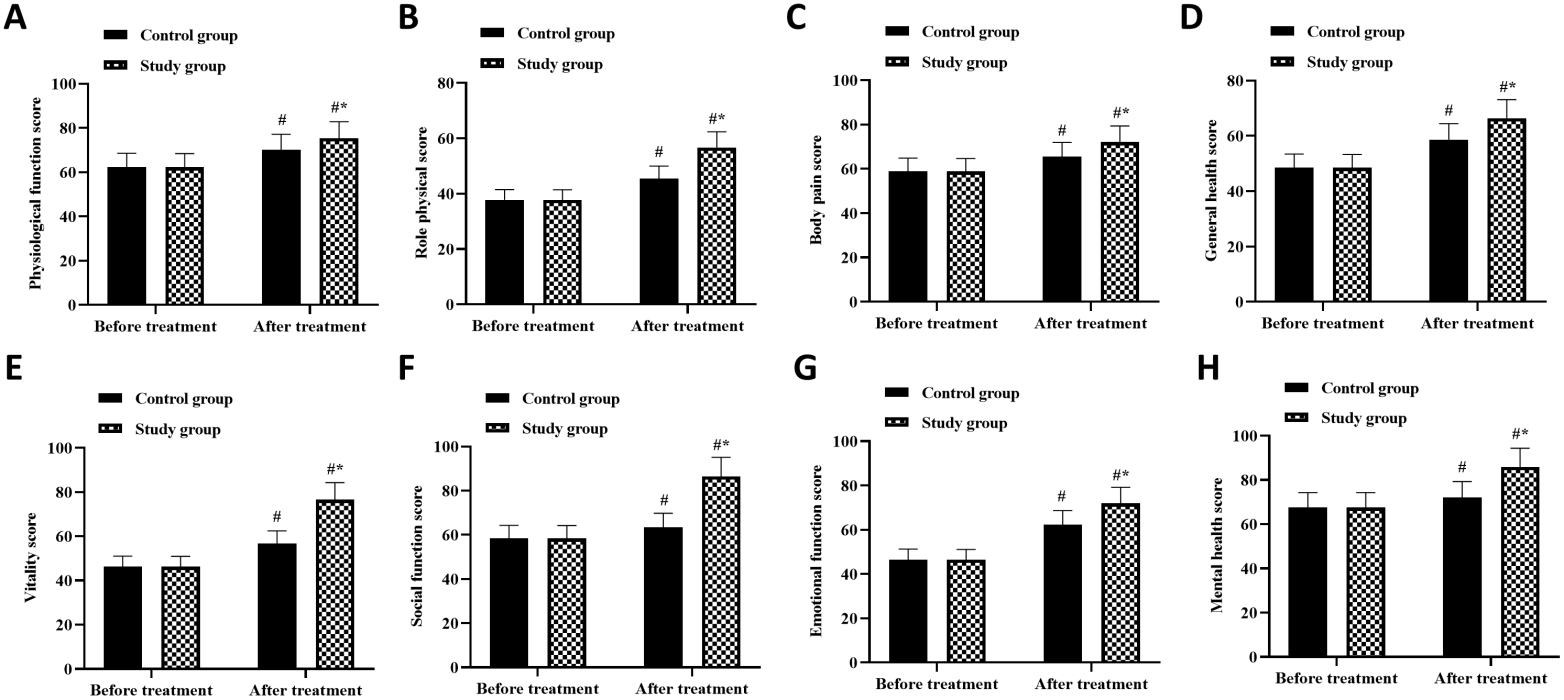
Health-related quality of life outcomes measured by SF-36 in Women with chronic pelvic pain. Comprehensive assessment of quality of life across eight domains of the Medical Outcomes Study 36-Item Short Form Health Survey (SF-36) comparing control group (CG) and study group (SG) at baseline and after 3 months of treatment. The eight domains include: **(A)** physical functioning (PF), **(B)** role-physical (RP), **(C)** bodily pain (BP), **(D)** general health (GH), **(E)** vitality (VT), **(F)** social functioning (SF), **(G)** role-emotional (RE), and **(H)** mental health (MH). Scores range from 0 to 100, with higher scores indicating better health status. Data are shown as mean values (*n* = 55 per group). ^#^*p* < 0.05 compared with baseline within the same group (paired *t*-test). ^*^p < 0.05 compared with control group at the same time point (independent *t*-test).

Health-related quality of life assessments demonstrated comprehensive improvements across physical and mental health domains, with superior outcomes in the combined therapy cohort (Figures 5A–H). The SF-36 physical component summary score increased from 42.3 ± 8.1 to 74.6 ± 9.3 in the study group, a 32.3-point improvement, compared to an increase from 41.9 ± 8.3 to 62.4 ± 8.7 in controls, a 20.5-point improvement (between-group *p* < 0.05). Mental component summary scores showed parallel patterns, improving from 45.6 ± 7.9 to 76.2 ± 8.4 in the study group versus 45.2 ± 8.1 to 65.3 ± 8.9 in controls (*p* < 0.05).

Domain-specific analysis revealed particularly robust improvements in bodily pain scores, which increased by 38.2 points with combined therapy versus 24.6 points with conventional therapy alone. Physical functioning and role-physical domains showed 35.4 and 33.7-point improvements, respectively, in the study group, compared to 22.1 and 21.3-point improvements in controls. Mental health domains demonstrated similar differential responses, with vitality scores improving by 31.8 points in the study group versus 19.4 points in controls, and social functioning improving by 34.2 versus 20.8 points, respectively. These multidimensional improvements suggest that combined therapy addresses not only physical symptoms but also enhances psychological well-being and social participation in women with chronic pelvic pain.

### Safety and tolerability

Both treatment approaches demonstrated acceptable safety profiles with no serious adverse events reported during the study period. In the study group, mild adverse events related to acupuncture included transient needle site discomfort in 4 patients (7.3%), minor bruising in 2 patients (3.6%), and mild dizziness following treatment in 1 patient (1.8%), all resolving spontaneously without intervention. Medication-related adverse events were comparable between groups, with gastrointestinal discomfort reported by 6 patients (10.9%) in the study group and 7 patients (12.7%) in the control group. No patients discontinued treatment due to adverse events, and all reported effects were classified as mild and self-limiting.

## Discussion

This prospective observational cohort study suggests that combining acupuncture with conventional pharmacotherapy may provide superior therapeutic outcomes compared to conventional treatment alone in women with CPP. The combined approach achieved a 94.55% overall response rate versus 78.18% with conventional therapy alone, with particularly notable improvements in pain intensity (45% greater VAS reduction), inflammatory markers (approximately 60% greater reduction in cytokines), sleep quality, and multiple quality of life domains. These findings suggest that integrated treatment addresses CPP through complementary mechanisms that enhance overall therapeutic efficacy.

The observed 16.37% absolute improvement in treatment response rate with combined therapy aligns with the growing evidence supporting multimodal approaches for chronic pain conditions (15), likely via synergistic analgesic mechanisms. While higher acetaminophen doses (e.g., 1 g) are used in some pain contexts, our lower-dose regimen prioritized long-term tolerability; future studies could evaluate acupuncture as add-on after failure of higher-dose standard therapy. The superiority of combined treatment probably reflects the distinct but complementary mechanisms of action. While conventional pharmacotherapy with tinidazole and acetaminophen addresses infection and provides analgesic effects, acupuncture appears to offer additional benefits through neuromodulation and enhanced anti-inflammatory activity (16). Acupuncture-induced analgesia involves multiple pathways including activation of descending inhibitory systems, release of endogenous opioids, and modulation of pain processing at spinal and cortical levels (17). The stimulation of specific acupoints used in our protocol (Sanyinjiao, Zusanli, Guilai, Zhongji, Guanyuan, and Qihai) has been shown to activate deep tissue receptors, alter pain thresholds, and reduce afferent pain signal transmission (17). Additionally, electroacupuncture at 2–100 Hz frequency, as used in our protocol, optimally activates different opioid receptor subtypes, with low frequencies primarily releasing enkephalins and β-endorphins, while high frequencies release dynorphins (18). This frequency-dependent mechanism may explain the robust analgesic effects observed.

The pronounced reduction in inflammatory cytokines with combined therapy provides mechanistic insight into the enhanced therapeutic response. IL-6, TNF-α, and IL-1β are key mediators in chronic pain pathophysiology (19). IL-6, produced by macrophages and spinal microglia, correlates with inflammation severity (20). TNF-α sensitizes nociceptors and enhances pain signal transmission (21), while IL-1β both directly sensitizes pain receptors and indirectly enhances prostaglandin synthesis (22). The approximately 60% reduction in inflammatory markers with combined therapy, compared to 32–37% with conventional treatment alone, suggests that acupuncture provides additional anti-inflammatory effects beyond those achieved with pharmacotherapy. This finding corroborates previous research showing acupuncture-mediated reduction of IL-6 and TNF-α in postmenopausal osteoporosis-related pain (23). The anti-inflammatory mechanism may involve acupuncture-induced increases in immunoglobulins and complement C3, enhancing local immune function and microcirculation (18).

The improvement in sleep quality, with 72.7% of combined therapy patients achieving normal PSQI scores compared to 38.2% in controls, represents a clinically meaningful benefit. Sleep disturbances and chronic pain often exhibit bidirectional relationships, with each condition exacerbating the other. The superior sleep outcomes with combined therapy may result from both direct pain reduction and acupuncture’s regulatory effects on circadian rhythms and autonomic function (24). Quality of life improvements across all SF-36 domains, particularly the 38.2-point improvement in bodily pain scores with combined therapy, demonstrate comprehensive treatment benefits extending beyond symptom relief. These findings align with previous studies showing acupuncture combined with conventional therapy enhancing quality of life in CPP patients (25). The improvements in mental health domains suggest that combined therapy addresses the psychological burden of chronic pain, potentially through modulation of neurotransmitter systems affecting mood and stress response.

In conclusion, combining acupuncture with conventional pharmacotherapy provides superior outcomes compared to conventional treatment alone in women with chronic pelvic pain, with significant improvements in pain intensity, inflammatory markers, sleep quality, and overall quality of life. The integrated approach demonstrated a favorable safety profile with enhanced therapeutic efficacy across multiple outcome domains. Several limitations should be considered when interpreting these findings. The observational, non-randomized design with patient self-selection may have introduced expectation or placebo effects, particularly for subjective outcomes such as VAS pain scores, PSQI, and SF-36. The absence of blinding further increases the risk of bias in patient-reported measures. Additionally, the study was conducted in a single center in China with a highly standardized acupuncture protocol, which may limit generalizability to other settings or less standardized practices. However, the objective reductions in inflammatory biomarkers (IL-6, IL-1β, TNF-α) provide biological plausibility and partially mitigate concerns related to subjectivity of the primary outcome. Future directions could include evaluating acupuncture as an add-on therapy specifically in patients who have failed standard higher-dose conventional treatments (e.g., 1 g acetaminophen regimens). These findings support incorporation of acupuncture into multimodal CPP management strategies, though randomized controlled trials are needed to confirm these observational findings and optimize treatment protocols. The substantial improvements observed suggest that addressing CPP through complementary mechanisms; combining pharmacological anti-inflammatory and analgesic effects with acupuncture-mediated neuromodulation; represents a promising therapeutic strategy worthy of broader clinical implementation and further investigation.

## Data Availability

The original contributions presented in the study are included in the article/supplementary material, further inquiries can be directed to the corresponding author.
